# Historical Portrayal of Hoarding Disorder in European Literature and Its Relationship to the Economic and Personal Circumstances of the Authors

**DOI:** 10.7759/cureus.31025

**Published:** 2022-11-02

**Authors:** Ryan A Chang, Vashisht Sekar

**Affiliations:** 1 History, The Harker School, San Jose, USA; 2 Department of Neurosurgery, Stanford University School of Medicine, Stanford, USA

**Keywords:** socioeconomic, hoarding, history, fictional literature, behavior

## Abstract

In 2013, hoarding disorder was officially recognized as a separate Diagnostic and Statistical Manual psychiatric diagnosis after years of debate. Prior to 2013, hoarding disorder was generally considered a subset of obsessive-compulsive disorder. Though modern medicine has only recently deepened the analysis of hoarding disorder, hoarding was regularly featured as a character trait in numerous European literary works dating back over 700 years. Several prominent European writers incorporated hoarding behavior in the fictional characters they created. Each author’s individual social and economic experiences may have been motivators for perpetuating hoarding-like behavior. It can be postulated that specific historical events and economic circumstances in the country at the time of each author’s life likely impacted their interpretation of hoarding behaviors, and the authors carried these influences into their portrayal of their fictional characters. This analysis discusses the various portrayals of hoarding in key pieces of literature and seeks to explain the rationale for these authors’ inclusion of hoarding traits in their characters.

## Introduction and background

Hoarding disorder is a mental disorder characterized by the significant accumulation of material goods, either as a result of acquiring excessive materials or failing to dispose of goods no longer needed [1]. In this diagnosis, the volume of collected material exceeds any logical rationale. Similar to other medical disorders, hoarding disorder can cause significant disruption in the life of the person afflicted as well as to their associated family members. Individuals with hoarding disorder can undergo significant stress if they fail to accumulate items and tremendous anguish with attempts to dispose of these possessions. The overall prevalence of hoarding disorder is approximately 4.0-5.8%, with higher rates observed in people over the age of 60 [2,3]. Hoarding is also associated with alcohol dependence, personality disorder traits, and specific childhood adversities [3]. Additionally, there does not appear to be a difference between men and women with respect to hoarding prevalence [2]. Prior to 2013, it was classified with obsessive-compulsive disorder (OCD) [4], and attempts to diagnose hoarding disorder involved applying standardized OCD testing to patients with compulsive hoarding behaviors.

Beginning in the 1990s, several medical researchers noticed that there appeared to be distinct differences between OCD and hoarding disorder, specifically that hoarding symptoms were not associated with the severe anxiety, stress, and negative affect typically associated with OCD [5-7] and that patients with hoarding disorder had a different onset and course of their disorder than patients with OCD [8]. In 2013, the fifth edition of the Diagnostic and Statistical Manual of Mental Disorders (DSM-5) was finalized, and this update separated hoarding disorder from OCD, classifying compulsive hoarding as a unique disorder [8].

While the medical field’s acknowledgment of hoarding disorder as a distinct diagnosis was only recent, European fictional literature has, for centuries, showcased multiple occurrences of hoarding traits in their characters. Literary writers tended to be highly educated, with access to and insights across diverse geographic regions and socioeconomic levels. Therefore, the observation that compulsive hoarding appears across multiple literary genres and countries of origin speaks to the view that hoarding was a behavior that was not only known but also of enough interest to be reflected in literature. Several hypotheses can explain the authors’ fictional characterization of hoarding in their literary characters. Personal upbringing, family experiences, or the presence of compulsive hoarding within the lives of the authors themselves may have provided fodder for introducing hoarding traits in their characters. Additionally, the economic circumstances before or at the time of the creation of these literary works may have impacted the authors’ perception of hoarding as a necessary, normal behavior. Evaluation of major European literary works by key authors (Dante, Shakespeare, Gogol, Balzac, and Dickens) and the corresponding backgrounds of each author reveal possible motivations for the inclusion of characters with hoarding behaviors in these works. The portrayal of hoarding as a human character trait will also be discussed.

The Divine Comedy, an epic poem written by Dante Alighieri, was believed to be written between 1308 and 1320. It is widely considered to be one of the premier works by an Italian author and was notable at the time for being written in the vernacular, whereas nearly all other works were published in Latin [9]. As a result, The Divine Comedy could be read by a larger portion of the population at the time, as most Italians could not read Latin. In Inferno, the first of three parts of The Divine Comedy, hoarders are considered sinners and are allocated to the Fourth Circle of Hell, where they spend their days in eternal conflict with wasters, smashing great weights (often depicted as large bags full of heavy coins) against each other. The hoarders scream at the wasters, “Why do you waste?” only to receive in reply the response, “Why do you hoard?” As the weights are equal to one another, the hoarders and the wasters are locked in an eternal battle that neither can win. Overseeing both the hoarders and the wasters is Pluto, the god of the underworld, also considered the Roman god of wealth. The hoarders and wasters are in hell because neither group was able to allocate money in moderation.

In William Shakespeare’s tragedy Coriolanus penned in 1608, the character Caius Marcius is a leader of a group of soldiers and politicians who hoard grain [10]. The people of Rome, suffering from famine and artificially inflated grain prices, plead with Caius for food from his stores. Caius refuses to apportion the grain, as he believes the common people are unworthy of these food supplies and only those with military service are honorable and deserving. Caius and the Roman Senate openly acknowledge that their grain hoarding increased the price of food for the populace, which, in turn, enriched their own wealth by inflating the value of their grain stores.

Nikolai Gogol wrote Dead Souls in 1842, a time period in Imperial Russia in which serfs were considered possessions who could be bought or sold. Wealthy landowners measured their net worth in part by the number of serfs they owned and often kept “dead serfs” on their estate records to inflate their wealth [11]. In Dead Souls, the protagonist Chichikov meets Plyushkin, one such wealthy landowner, and soon realizes that Plyushkin accumulates every material item he comes across. The local people nickname him the “fisherman” due to his compulsion to “catch and keep” these items. After Chichikov consummates a deal with Plyushkin to buy some of his “dead serfs,” Plyushkin instructs one of his servants to locate an old cake left over from years ago, scrape off the mold, and serve it to Chichikov. Because he eventually found use for this old cake, Plyushkin justifies his hoarding behavior, gloating that his decision to keep this cake was correct.

Le Cousin Pons was one of the last novellas written by Honoré de Balzac in 1846 as part of his Comedie Humanie, a series of related stories during the time period of the French Restoration after Napoleon’s downfall [12]. Pons, the primary character in the novella, is a musician and songwriter who works during the day as a conductor in an orchestra. During his youth, and as a result of his tours of Europe during his musical training, he acquired a taste for art and antiques and subsequently spent his adult life with an abnormal desire to collect these material items. His focus was to identify and acquire pieces of relatively low value in the hopes that they would one day be worth a fortune. In the novella, Pons’ collection reaches a museum-level quality, but despite this, he cannot bear to sell a single piece of the collection. He refuses to seek out buyers for his items despite becoming impoverished and struggling to make a living from his day job. In the end, Pons dies with his collection intact, never benefitting from it.

Bleak House, written by Charles Dickens in 1862, is largely believed to be a satire on the English judicial system [13]. Much of the novel focuses on attempts to resolve a probate court case that has largely exhausted the funds within an estate due to decades of legal challenges. In the novel, Krook, a shop owner working in London, acquires numerous material possessions, with a preference for legal documents. Krook’s store lists many items he is seeking to purchase, but while Krook is willing to buy items, he offers nothing for sale. Krook’s faulty logic reveals that while he acquires items with the intent to sell them for future gain, his compulsive hoarding precludes him from ever turning a profit. Ironically, buried among all the papers that Krook hoards are pages with key facts that would allow the probate case to be resolved. Krook’s massive hoarding of documents and failure to organize or release any subsequently leads to the decades-long duration of the probate.

## Review

In light of detailed descriptions of hoarding in literature for centuries, it is interesting to explore why the medical field did not consider hoarding disorder a distinct DSM diagnosis until 2013. These five European literary classics across multiple centuries portray hoarding with similar behavioral characteristics. All of these hoarding characters are depicted as continual accumulators who cannot part with their possessions. On a broader level, it is possible that the personal circumstances of each author and the economic conditions of the author’s time period made the collection of items normal behavior.

Dante’s Inferno was written in part as a criticism of the Catholic Church of Dante’s time [14]. He viewed popes and cardinals of the Church as miserly individuals who did not put the teachings of the Church into practice but instead used their offices to enrich themselves. In the Fourth Circle of Hell in Inferno, the hoarders are often depicted as having a monk’s haircut, implying that the Church and its leaders were guilty of material accumulation. In this context, hoarding wealth exemplified the character flaw of greed. Dante classified hoarding as a sin worse than lust and gluttony, both of which were located on more superficial levels of Hell and thus not as egregious as greed. Dante’s Catholic upbringing instilled in him an initial strong belief in the Church, but the corruption and simony he observed in the leaders of the Church, particularly during the papacy of Boniface VIII (1297-1303), convinced him that the Church had lost its way. Dante expected Church leaders to have an obligation to respond to a higher calling and serve as role models for the common people. His disillusionment with the Church grew as he realized that the non-religious people of his time appeared to be less sinful than Church leaders. As a result, he no longer viewed the popes as being infallible and used Inferno as a platform to criticize them [15]. Given that corruption and greed were so widespread within the Church during Dante’s time [14], hoarding as depicted in Inferno likely was not considered unusual or abnormal. Instead, this hoarding behavior was regarded more as a sin of greed rather than a peculiar medical disorder.

William Shakespeare’s characterization of grain hoarding in Coriolanus was possibly shaped by his own personal experiences in England a decade before he wrote the play [16]. The Black Death plague of 1592-1593 heralded several years of poor harvest throughout most of Europe, resulting in the starvation of the populace during the most severe famine in England in nearly half a century [17]. While Shakespeare is best known as a playwright, nearly all his earnings during the late 16th century were obtained outside of writing. Interestingly, much of his wealth resulted from his hoarding of grain during years of surplus and later selling the grain during years of famine at significantly increased prices [18]. In this case, Shakespeare hoarded for capitalistic purposes, and his depiction of Caius Marcius in Coriolanus appears to mirror Shakespeare’s real-life behavior. Shakespeare was also a money lender, and he considered hoarding for future sale at higher prices an accepted method to earn income. Shakespeare was not the only hoarder and money lender, as a number of similar businesses were in practice around London for centuries before Shakespeare’s time [19]. Therefore, hoarding behavior in Shakespeare’s time may have been overlooked as prudent planning rather than a medical disorder.

Nikolai Gogol lived during a time of great suffering for the Russian serfs with the harsh geographic climate and absolute monarchist government locking them into poverty. Famine was frequent, and the severe Russian winters made storage of food stocks not only wise but essential for survival. By the late 1700s, the Imperial Russian government had even passed laws requiring landowners to set aside grain for later use in attempts to mitigate hunger [20]. While Gogol was not a peasant, he witnessed firsthand the value of setting aside food or items that could be later exchanged for food [21]. Dead Souls was written as a social satire, and Plyushkin represents the comedic extreme of such hoarding for the future. In Plyushkin’s case, hoarding encompasses even non-useful items such as old rags, iron nails, and broken pottery [9]. While hoarding such useless items provides background comedy, Gogol perpetuates the view that hoarding may have been driven by practical common sense in building food reserves, obscuring it as a mental disorder. Plyushkin’s character is so well known in Russian literature that even today people who have an obsession to collect items are considered to have “Plyushkin’s syndrome” [22].

Honoré de Balzac’s character Pons hoards antiques and art, items that have significant intrinsic value [12]. Pons appears to mimic Balzac’s own life, as Balzac exhibited hoarding in his attempt to earn the hand of Countess Eveline de Hanska for nearly two decades. To convince her to relocate from her home country of Ukraine to France, Balzac purchased a house on the rue Fortunée for 50,000 francs (despite being 200,000 francs in debt at the time) and stocked it with antique furniture, artworks, rare china and silver, and various other ostentatious items collected from all over Europe [23]. The countess delayed her marriage to Balzac because her family did not approve of him, and she was embroiled in a court case regarding the death of her first husband. During this delay, Balzac continued to spend a greater and greater fortune accumulating items in his home under the belief that these material items would buy her affection. Similar to Pons, Balzac’s life has a tragic ending. Five months after the countess finally agreed to marry him, Balzac died from complications of gangrene in their lavishly furnished home [23]. Le Cousin Pons was written during the period of time when Balzac was actively wooing the countess, suggesting that his personal life greatly influenced his characterization of Pons. Balzac goes to great lengths to rationalize his own hoarding as simply requisite outfitting of a residence befitting his countess. Similarly, Balzac imbued his literary character with compulsive desires to acquire items under the pretense that it will net him a big gain eventually. And in a sad irony, like Pons, Balzac dies surrounded by his museum-level possessions, but without the end goal that he had sought with them. Thus, in Balzac’s time, his hoarding behavior could have been shrouded as a courtship quirk rather than symptomatic of a clinical disorder.

Charles Dickens experienced firsthand the effects of debt and poverty as a child [24]. His education was cut short when both of his parents were imprisoned for failing to pay their debts, and this trauma of being turned out onto the streets lasted throughout Dickens’ lifetime. He was forced to take low-paying jobs, and this impacted his view of the need for socioeconomic reforms to help the poor [24]. Similar to Gogol, Dickens’ view of hoarding was that it was a normal, necessary behavior for economically disadvantaged individuals to survive. In the case of Bleak House, hoarding was portrayed as satire, as the hoarder Krook actually has the key information that would resolve the probate case that serves as the basis for the novel. The fact that this information is buried in the collection of Krook’s legal documents and not available to the barristers who are trialing the case only adds to the humor. The fact that Krook runs a trading shop (though trades appear to only occur in one direction) provides his justification to collect esoteric items. Though Dickens satires extreme hoarding, he acknowledges that the fastidious behavior of the poor to collect and hold onto items was a consequence of survival rather than a medical disorder [25].

Collectively, these fictional European literary works depict characters who, from a modern perspective, show behavior consistent with hoarding. However, when the circumstances of the authors are analyzed, a great deal of the behavior appears to be justified by the economic conditions of their time, making it difficult to visualize these behaviors as a medical disorder. Shakespeare and Gogol both experienced significant episodes of famine in the course of their lives, so during their time periods, individuals who amassed food stocks would have been considered as having great foresight rather than suffering from a medical disorder. Dickens lived through poverty while young, ingraining in him a belief that maintaining a reserve of both money and food was essential. In these situations, the economic need made it difficult to classify the acquisition of items as a medical disorder in which the collection of items is done for no real practical purpose. In other works, hoarding of wealth was depicted as part of a social commentary. Dante portrayed the accumulation of wealth among church leaders as so widespread and such common behavior that it was never considered pathologic. Pons’ collection of valuables mirrored Balzac’s own collection, which he justified as a necessary expense to climb the social ladder from the French bourgeoisie to the upper class, and this obscured any inkling of a medical disorder.

The modern DSM-5 diagnosis of hoarding disorder has clearly defined parameters [26]. These parameters include (1) persistent difficulty discarding or parting with possessions, regardless of their actual value; (2) the difficulty is due to a perceived need to save the items and to the distress associated with discarding them; (3) the difficulty discarding possessions results in the accumulation of possessions that congest and clutter active living areas and substantially compromises their intended use; (4) the hoarding causes clinically significant distress or impairment in social, occupational, or other important areas of functioning; (5) the hoarding is not attributable to another medical condition; and (6) the hoarding is not better explained by the symptoms of another mental disorder. Because hoarding often manifests as an intriguing behavior, it often was used in European literature to attract the reader and provide interesting character development. However, taking into consideration the personal and economic circumstances of the authors, a rationalization for these behaviors can be conceived. In many cases, what currently would be considered hoarding behavior would not have received the same level of scrutiny in previous times. People with true hoarding disorders would have been more difficult to segregate from those who were accumulating items for more practical reasons.

## Conclusions

The depiction of hoarding in European literary works crosses countries and genres over many centuries. While much of the present-day focus on hoarding is largely confined to psychiatric interpretations as a result of the new DSM-5 classification, historical portrayals of hoarding have involved behaviors that are a direct response either to the personal experiences of the authors or the economic circumstances of the author’s time period. Contrary to the refinements of the present, the survival challenges of the past often necessitated storing up food and supplies to ensure sufficient future security. Neither these classical European writers nor their medical contemporaries appear to consider hoarding a medical disorder during their time, but rather a prudent response to the economic circumstances in which most people lived. Hoarding was misjudged from this standpoint until the present when hoarding was seen in situations with a lack of a true economic, historic, or cultural reason, helping to focus attention on this visible disorder.
